# Azilsartan ameliorates ox-LDL-induced endothelial dysfunction via promoting the expression of KLF2

**DOI:** 10.18632/aging.202973

**Published:** 2021-05-04

**Authors:** Wenfeng Li, Chenggao Wang, Dandan Zhang, Kanghua Zeng, Shihui Xiao, Feng Chen, Jun Luo

**Affiliations:** 1 Department of Cardiology, The First Affiliated Hospital of Jinan University, Guangzhou 510630, Guangdong, China; 2 Department of Cardiology, Ganzhou People’s Hospital, Ganzhou 341000, Jiangxi, China

**Keywords:** Azilsartan, ox-LDL, endothelial dysfunction, atherosclerosis

## Abstract

Background: Oxidized LDL(Ox-LDL) mediated endothelial dysfunction is involved in the pathogenesis of various cardiovascular diseases, including atherosclerosis. Azilsartan is a potent agent for the treatment of hypertension as the antagonist of the angiotensin II receptor. This study will investigate whether Azilsartan possesses a beneficial effect against endothelial cell dysfunction induced by ox-LDL and explore the underlying preliminary mechanism.

Methods: Ox-LDL was applied to construct an *in vitro* endothelial dysfunction model in human umbilical vascular endothelial cells (HUVECs). The expression of lectin-type oxidized LDL receptor 1 (LOX-1), endothelial nitric oxide synthase (eNOS), tight junction protein occludin, and transcriptional factor Krüppel-like factor 2 (KLF2) was detected using qRT-PCR and Western blot. ELISA and qRT-PCR were utilized to evaluate the production of chemokine monocyte chemotactic protein 1 (MCP-1) and chemokine (C-X-C motif) Ligand 1 Protein (CXCL1) in treated HUVECs. The generation of nitro oxide (NO) was determined using DAF-FM DA staining assay. KLF2 was silenced by transfecting the cells with specific Small interfering RNA (siRNA). FITC-dextran permeation assay was used to check the endothelial monolayer permeability of treated HUVECs.

Results: Firstly, the elevated expressions of LOX-1, MCP-1, and CXCL-1 induced by stimulation with ox-LDL were significantly suppressed by Azilsartan. The downregulated eNOS and reduced production of NO induced by ox-LDL were reversed by the introduction of Azilsartan. Secondly, enlarged endothelial monolayer permeability and decreased expression of occludin stimulated with ox-LDL were greatly reversed by treatment with Azilsartan but were abolished by silencing the expression of KLF2. Lastly, the inhibited expression of KLF2 induced by ox-LDL was significantly elevated by the introduction of Azilsartan.

Conclusion: Azilsartan might ameliorate ox-LDL-induced endothelial damage via elevating the expression of KLF2.

## INTRODUCTION

High morbidity due to cardiovascular diseases has been reported as the major cause of disability and death in China [1] and is responsible for approximately 40% of the total deaths annually [1, 2]. It is reported that atherosclerosis (AS) is the pathological basis of most cardiovascular diseases and is mainly induced by the dysfunction of endothelial cells located in the inner wall of blood vessels, the adhesion of monocytes, and the migration and proliferation of smooth muscle cells [3, 4]. The inflammation induced by endothelial dysfunction is regarded as the initial step of AS and, the adhesion between vascular endothelial cells and monocytes or leukocytes mediated by a series of adhesion molecules is reported to be the early characteristic and key step of AS [5]. In pro-atherogenic conditions, circulating low-density lipoprotein (LDL) cholesterol is prone to be oxidized by oxygen-free radicals. The recruited monocytes in the lipid-rich subendothelial space of affected arteries could become macrophages capable of taking up oxidized LDL (ox-LDL). Ox-LDL stimulates the secretion of adhesion molecules and chemokines, such as ICAM-1, VCAM-1, and CCL-2 in endothelial cells, which contribute to the migration and adhesion of leukocytes [6]. Ox-LDL functions through binding to several scavenger receptors (SRs), including SR-A, SR-BI, CD36, and LOX-1 [7], the expressions of which can be upregulated by stimulation with ox-LDL [8]. Activated LOX-1 in turn upregulates the expression of pro-inflammatory cytokines and chemokines, including IL-8, CXCL2, and CXCL3 [9]. In addition to adhesion molecules and chemokines, endothelial dysfunction can also be induced by the accumulation of hyperoxides and the reduction of the synthesis of nitric oxide (NO), which is called vascular oxidative stress. Consequently, continuous endothelial inflammation is triggered by oxidative stress and further accelerates the development of AS [10]. With the development of endothelial dysfunction, the hyperpermeability of endothelial monolayer is induced by stimulation with ox-LDL through impacting the expression of cellular tight junction proteins, such as occludin [11, 12]. It is currently reported that Kruppel-like factor 2 (KLF2), an important transcriptional regulator downregulated by ox-LDL, is regarded as an important regulator of endothelial function. KLF2 possesses a variety of physiological functions, including the control of NO availability, regulation of vascular inflammation, and modulation of endothelial permeability [13, 14]. Therefore, KLF2 might be a promising target for the treatment of cardiovascular diseases.

**Figure 1:**
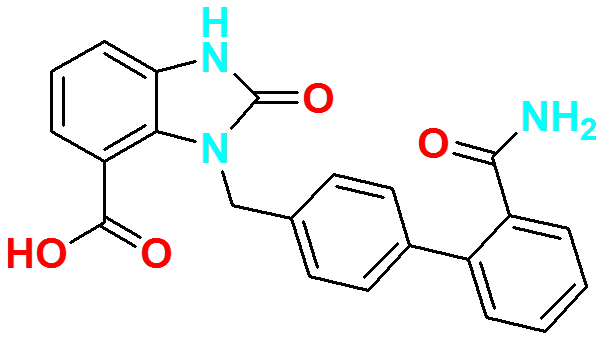
Molecular structure of Azilsartan.

Azilsartan (Figure 1), an agent developed by Takeda for the treatment of hypertension*,* is an antagonist of angiotensin II and acts in a dose-dependent manner [15]. Following the administration of Azilsartan to healthy subjects, the plasma concentration of angiotensin I and II are elevated greatly, accompanied by increased renin activity and decreased plasma aldosterone concentrations [16]. Recently, a significant protective property of Azilsartan against hydroperoxide induced oxidative stress in endothelial cells has been reported [17]. In the present study, the effects of Azilsartan on the ox-LDL-induced endothelial dysfunction, as well as the underlying mechanism, will be investigated to explore its potential therapeutic properties against cardiovascular diseases, such as AS.

## RESULTS

### The expression of LOX-1 in the HUVECs induced by ox-LDL was downregulated by Azilsartan

**Figure 2:**
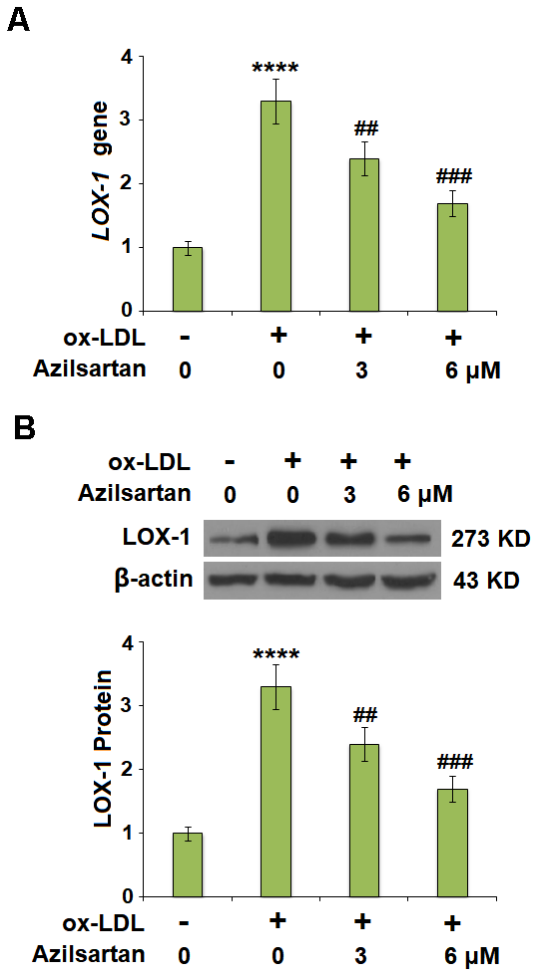
**Azilsartan reduced the expression of LOX-1 in HUVECs.** Cells were stimulated with ox-LDL (100 μg/mL) in the presence or absence of Azilsartan (3, 6 μM) for 24 hours. (**A**) Gene expression of LOX-1; (**B**) Protein of LOX-1 (****, P<0.0001 vs. vehicle group; ##, ###, P<0.01, 0.001 vs. ox-LDL group, N=6 for each group).

Previous studies have shown the exposure of 50-100 μg/ml ox-LDL for 24 hours induced significant endothelial cell injury in HUVCEs [18, 19], therefore we adopted 100 μg/ml as a treatment dose in our study. LOX-1 is the main receptor of ox-LDL and plays an important role in mediating its intracellular signaling. The cells were stimulated with ox-LDL (100 μg/mL) in the presence or absence of Azilsartan (3, 6 μM) for 24 hours and the expression of LOX-1 was evaluated using qRT-PCR and Western blot. As shown in Figure 2A, 2B, LOX-1 was significantly upregulated by stimulation with ox-LDL but was greatly downregulated by treatment with Azilsartan in a dose-dependent manner.

### Azilsartan prevented the ox-LDL-induced expressions of MCP-1 and CXCL1 in HUVECs

**Figure 3:**
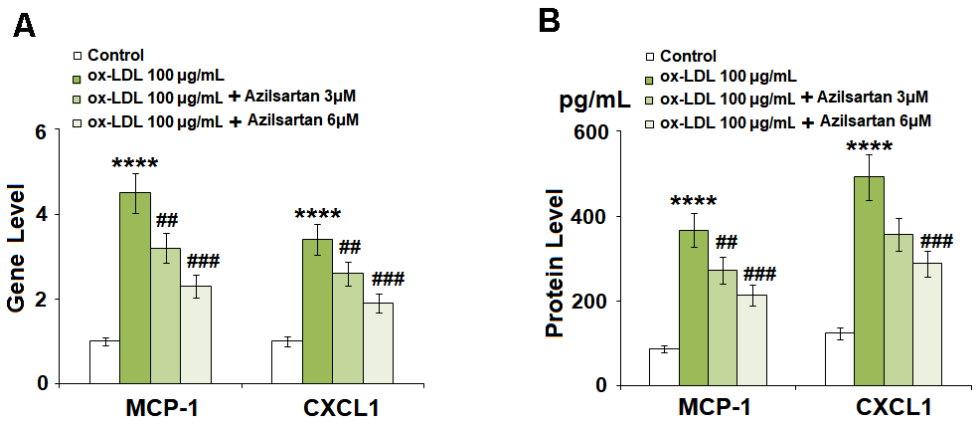
**Azilsartan prevented ox-LDL-induced expressions of MCP-1 and CXCL1 in HUVECs.** Cells were stimulated with ox-LDL (100 μg/mL) in the presence or absence of Azilsartan (3, 6 μM) for 24 hours. (**A**) Gene expressions of MCP-1 and CXCL1; (**B**) Secretions of MCP-1 and CXCL1 (****, P<0.0001 vs. vehicle group; ##, ###, P<0.01, 0.001 vs. ox-LDL group, N=5-6 for each group).

To evaluate the effects of Azilsartan on the production of adhesion molecules and chemokines, the concentrations of MCP-1 and CXCL1 secreted by the treated HUVECs were detected using qRT-PCR and ELISA assay. As shown in Figure 3A, the elevated gene expressions of MCP-1 and CXCL1 induced by stimulation with ox-LDL were significantly suppressed by treatment with Azilsartan in a dose-dependent manner. Figure 3B shows the protein concentrations of MCP-1 and CXCL1 determined using ELISA. The average concentrations of MCP-1 in the control, ox-LDL, 3 μM Azilsartan, and 6 μM Azilsartan group were 86.5, 366.8, 272.9, and 213.2 pg/mL, respectively. Furthermore, approximately 123.6, 491.8, 356.2, and 287.7 pg/mL CXCL1 were detected in the HUVECs treated with blank medium, ox-LDL, ox-LDL in the presence of 3 μM Azilsartan, and ox-LDL in the presence of 6 μM Azilsartan, respectively.

### Azilsartan ameliorated the ox-LDL-induced reduction of eNOS and nitric oxide

**Figure 4:**
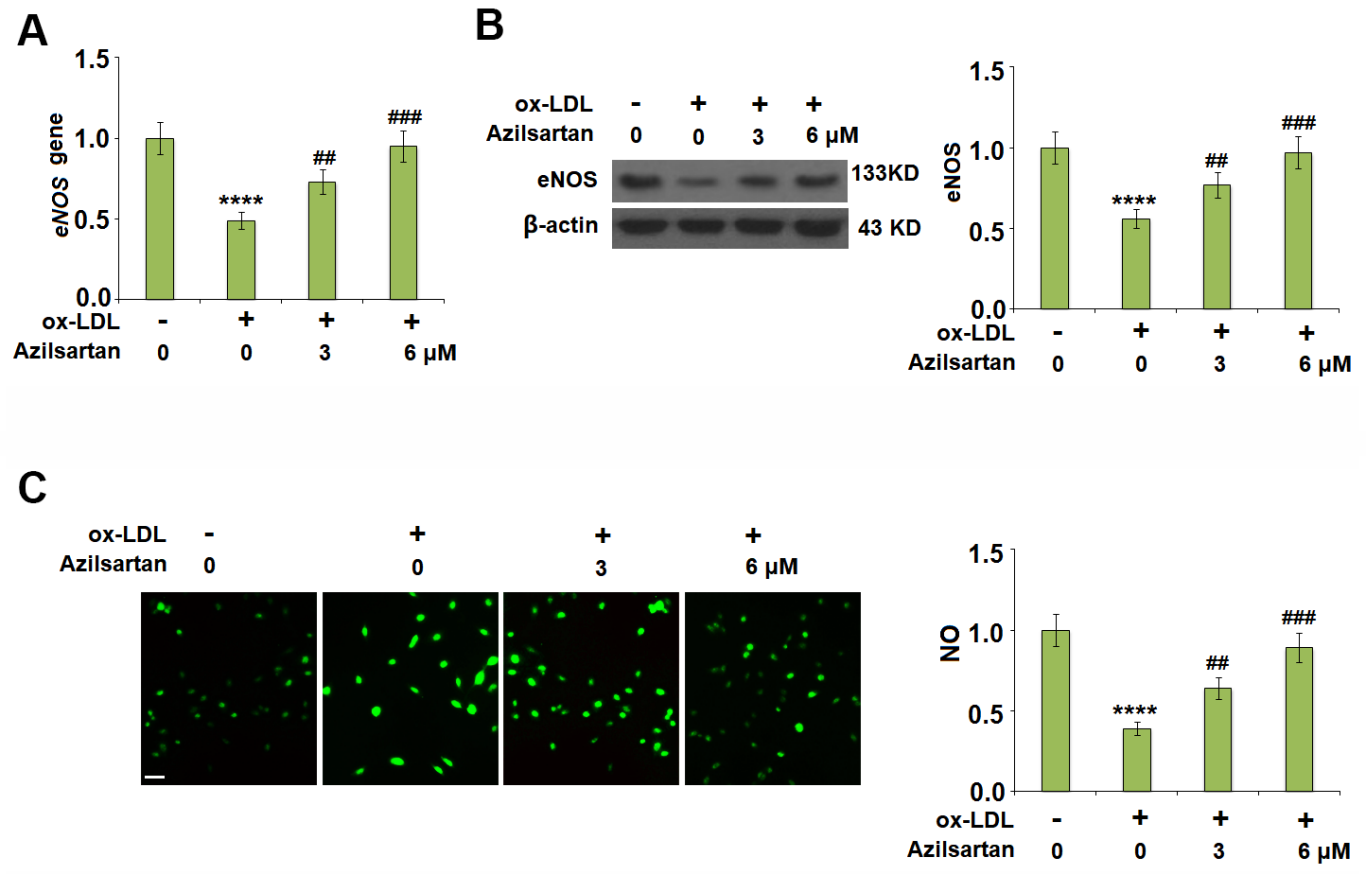
**Azilsartan ameliorated ox-LDL-induced reduction of eNOS and nitric oxide (NO).** Cells were stimulated with ox-LDL (100 μg/mL) in the presence or absence of Azilsartan (3, 6 μM) for 24 hours. (**A**) Gene expression of eNOS; (**B**) Protein of eNOS; (**C**) Production of NO. Scale bar, 200 μm (****, P<0.0001 vs. vehicle group; ##, ###, P<0.01, 0.001 vs. ox-LDL group, N=5-6 for each group).

To investigate the effects of Azilsartan on the endothelial oxidative stress induced by ox-LDL, the expression of eNOS and the production of NO were detected. As shown in Figure 4A, 4B, the expression of eNOS was significantly suppressed by stimulation with ox-LDL but was greatly elevated by the introduction of Azilsartan in a dose-dependent manner. In addition, the inhibited secretion of NO-induced by treatment with ox-LDL (Figure 4C) was significantly promoted by administration with Azilsartan in a dose-dependent manner.

### The enlarged endothelial monolayer permeability induced by ox-LDL was mitigated by Azilsartan

**Figure 5:**
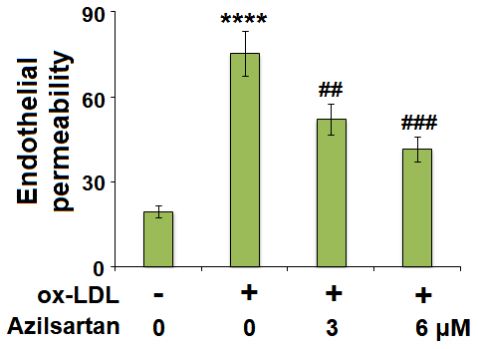
**Azilsartan mitigated ox-LDL-induced endothelial permeability of HUVECs.** Cells were stimulated with ox-LDL (100 μg/mL) in the presence or absence of Azilsartan (3, 6 μM) for 24 hours. Endothelial monolayer permeability was measured by FITC-dextran permeation (****, P<0.0001 vs. vehicle group; ##, ###, P<0.01, 0.001 vs. ox-LDL group, N=6 for each group).

**Figure 6:**
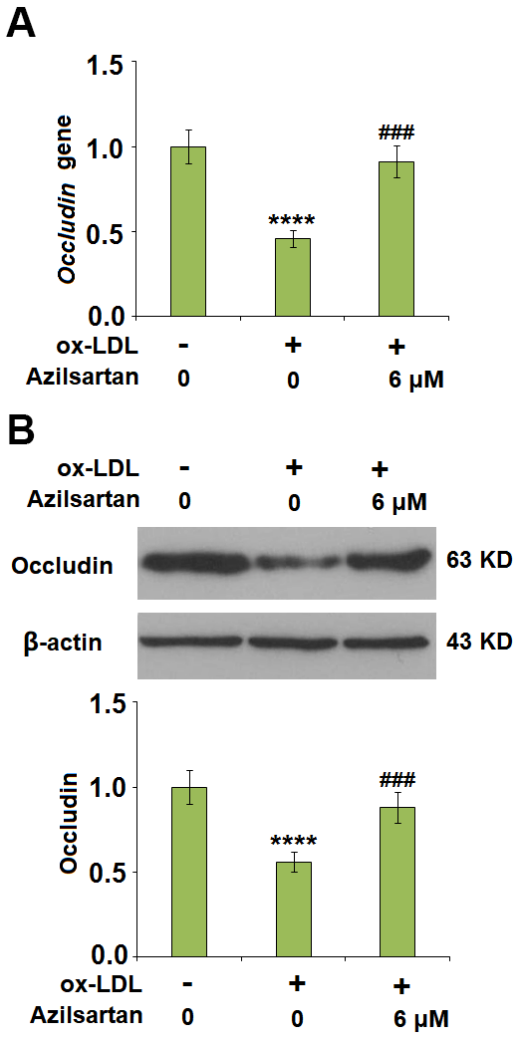
**Azilsartan mitigated ox-LDL-induced expression of occludin.** Cells were stimulated with ox-LDL (100 μg/mL) in the presence or absence of Azilsartan (6 μM) for 24 hours. (**A**) Gene expression of Occludin; (**B**) Protein of Occludin (****, P<0.0001 vs. vehicle group; ###, P<0.001 vs. ox-LDL group, N=6 for each group).

The endothelial monolayer permeability of HUVECs was evaluated using the FITC-dextran permeation assay. As shown in Figure 5, the endothelial monolayer permeability of HUVECs incubated with blank medium, ox-LDL, ox-LDL in the presence of 3 μM Azilsartan, and ox-LDL in the presence of 6 μM Azilsartan were 19.5%, 75.3%, 52.1%, and 41.6%, respectively. To further explore the mechanism, the expression level of occludin, a tight-junction protein, was determined. As shown in Figure 6, cells were stimulated with ox-LDL (100 μg/mL) in the presence or absence of Azilsartan (6 μM) for 24 hours. We found that occludin was significantly downregulated by stimulation with ox-LDL but was greatly reversed by treatment with Azilsartan, indicating a promising repairment of Azilsartan against injured tight junction.

### Azilsartan might regulate the occludin expression and endothelial permeability by mediating the expression level of KLF2

**Figure 7:**
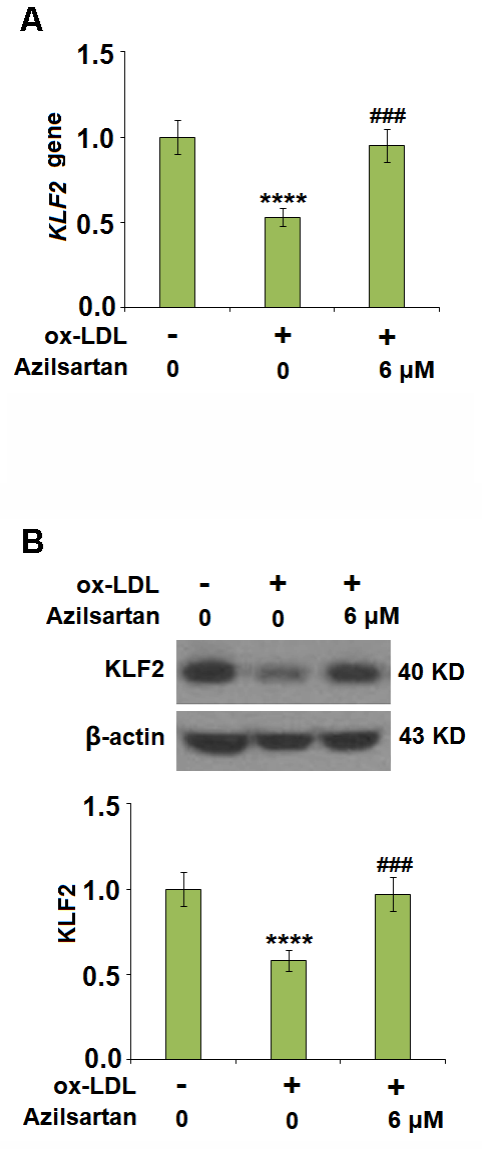
**Azilsartan restored ox-LDL-induced reduction of KLF2 in HUVECs.** Cells were stimulated with ox-LDL (100 μg/mL) in the presence or absence of Azilsartan (6 μM) for 24 hours. (**A**) Gene expression of KLF2; (**B**) Protein of KLF2 (****, P<0.0001 vs. vehicle group; ###, P<0.001 vs. ox-LDL group, N=6 for each group).

**Figure 8:**
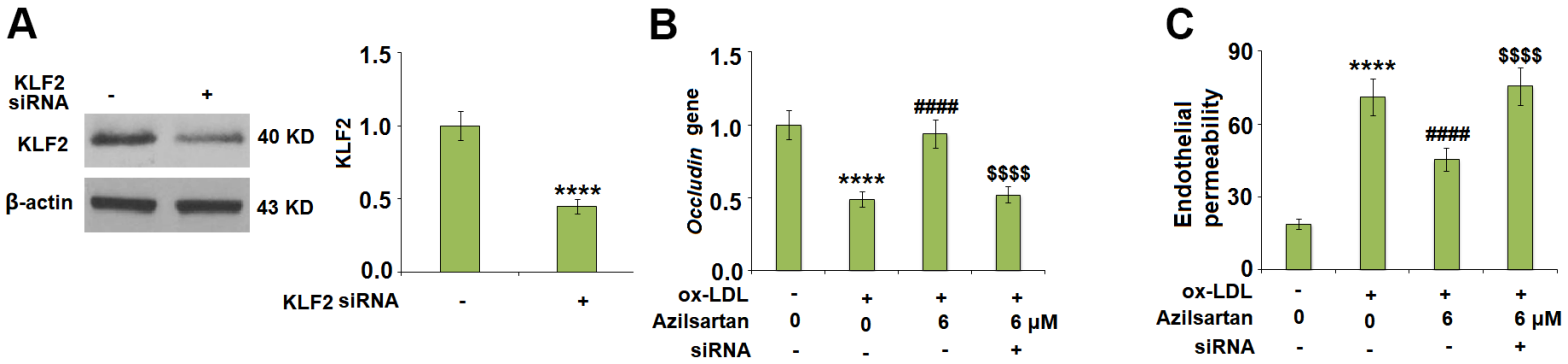
**Silencing of KLF2 abolished the effects of Azilsartan in occludin expression and endothelial permeability.** Cells were transfected with KLF2 siRNA, followed by stimulation with ox-LDL (100 μg/mL) in the presence or absence of Azilsartan (6 μM) for 24 hours. (**A**) Successful knockdown of KLF2 as measured by western blot analysis. (**B**) Gene expression of occludin; (**C**) Endothelial monolayer permeability (****, P<0.0001 vs. vehicle group; ####, P<0.0001 vs. ox-LDL group; $$$$, P<0.0001 vs. ox-LDL+ Azilsartan group, N=5-6 for each group).

To explore the potential molecular mechanism, we focused on the key regulator-KLF2. As shown in Figure 7, we found that the expression level of KLF2 in HUVECs was significantly suppressed by the introduction of ox-LDL but was greatly elevated by treatment with Azilsartan. To verify the function of KLF2 that mediates the effects of Azilsartan, we knocked down the expression of KLF2 by transfecting the HUVECs with siRNA against KLF2. Results in Figure 8A display the successful knockdown of KLF2. Subsequently, the transfected cells were stimulated with ox-LDL in the presence of Azilsartan. As shown in Figure 8B, the suppressed expression of occludin induced by ox-LDL was significantly elevated by treatment with Azilsartan but reversed by the knockdown of KLF2. Further, the endothelial monolayer permeability of HUVECs incubated with blank medium, ox-LDL, ox-LDL in the presence of Azilsartan and KLF2 siRNA were 18.8%, 71.2%, 45.3%, and 75.6%, respectively, indicating that the silencing of KLF2 abolished the effects of Azilsartan on occludin expression and endothelial permeability.

## DISCUSSION

AS is a chronic inflammatory vascular disease defined by lipid and inflammatory cell accumulates in the walls of arteries [20, 21]. When the balance between the production of ROS and the antioxidant defense system is broken, oxidative stress is induced and endothelial dysfunction triggered, which further contributes to the development of AS [22, 23]. Increased ROS results in the oxidation of native LDL to ox-LDL and is currently, widely used to simulate endothelial dysfunction *in vitro* model in HUVECs [24–26]. By interaction with LOX-1, ox-LDL suppresses the constitutive eNOS expression [27], elevates the expression of adhesion molecules and chemokines in endothelial cells, and induces macrophage proliferation, collagen production, smooth muscle cell migration, and platelet activation [28]. In the present study, we also used ox-LDL to induce endothelial dysfunction in HUVECs. By treatment with Azilsartan, these pathological changes caused by ox-LDL were reversed, indicating a potential protective effect of Azilsartan against endothelial dysfunction. However, the present study only provides a preliminary result, which needs more experimental data to support its conclusion. To further verify the protective property of Azilsartan against endothelial dysfunction, more adhesion molecules (such as VCAM-1 and ICAM-1) and chemokines (such as CXCL2 and CCL2), as well as inflammatory factors (such as IL-6, TNF-α) will be introduced into the analysis system in our future work. Also, functional experiments, such as macrophage proliferation assay, migration assay of smooth muscle cells, and apoptosis of endothelial cells will be added in our future work to better understand the potential therapeutic property of Azilsartan against cardiovascular diseases.

In the late 1970s, Minick et al [29, 30] proposed and verified that endothelium severe endothelial hyperpermeability contributes to the uptake of intimal lipids and a hypercholesterolemic rabbit model further confirmed that the labeled LDL mainly passed through the sites where inter-endothelial junctions were impaired [31]. Therefore, the endothelial permeability is of great significance in preventing the deposit of lipids within the vessels and is a key inducer for various cardiovascular diseases, including AS [32]. Endothelial cell-cell junction is one of the factors involved in the maintenance of endothelial permeability [33, 34]. Numerous and highly ordered junctional complexes between endothelial cells are observed in the early electron microscopy studies [35]. These junctional complexes include adherent junctions and tight junctions, such as occludin and claudin [36]. In the present study, we found that the endothelial monolayer permeability was significantly enlarged and the tight junction was significantly damaged by stimulation with ox-LDL but was greatly ameliorated by treatment with Azilsartan, indicating a potential protective effect of Azilsartan against injured tight junction and endothelial permeability. In our future work, functional experiments, such as the uptake of labeled ox-LDL, will be performed to further verify this property. KLF family is reported to maintain the integrity of the endothelial barrier function by promoting the promoter activity of tight junction-related proteins including ZO-1, occludin, and Claudin5 [37]. We found that the protective effect of Azilsartan against injured tight junction and endothelial permeability induced by ox-LDL was significantly abolished by downregulating the expression of KLF2, indicating that KLF2 might be an important mediator for the regulation of Azilsartan on endothelial permeability. In our future study, the direct or indirect interaction between Azilsartan and KLF2 will be further investigated to better understand the functional mechanism of Azilsartan on endothelial dysfunction and thereafter on cardiovascular diseases.

Taken together, our data show that Azilsartan might ameliorate ox-LDL-induced endothelial dysfunction via elevating the expression of KLF2.

## MATERIALS AND METHODS

### Cell culture and treatment

All the experiments were designed in accordance with the “World Medical Association Declaration of Helsinki Ethical Principles for Medical Research Involving Human Subjects”. The study was approved by the Ethical Committee of Ganzhou People’s Hospital (GZH-2017021). HUVECs were purchased from the Type Culture Collection of the Chinese Academy of Sciences (TCCCAS, Shanghai, China) and cultured with an EGM-2 Bullet Kit (Lonza, Switzerland) containing 0.1% gentamicin sulfate (GA-1000). HUVECs were routinely maintained in 2% low serum complete growth media EGM2 for less than seven passages, and all cultures were maintained in a humidified 37° C atmosphere. Azilsartan (98% purity) was purchased from Sigma-Aldrich, MO, USA (#SML0432), and Ox-LDL from Thermo Fisher Scientific, MA, USA (#L34357). For treatment experiments, HUVECs were plated on 35 or 60-mm dishes at a density of 2x10^4^ cells/cm^2^ area overnight, HUVECs were treated with ox-LDL (100 μg/ml) [18, 19] in the presence or absence of Azilsartan (3, 6 μM) [17] for 24 hours.

### Real-time PCR analysis

The TRIzol solution (Qiagen, Switzerland) was used to extract the total RNA from the HUVECs according to the instructions of the manufacturer, followed by transcribing the RNAs to cDNA using the SuperScript III (Takara, Tokyo, Japan) reagent. The SYBR green quantitative PCR kit (Takara, Tokyo, Japan) was used to determine the relative expression of target genes, while GAPDH was taken as the negative control. QRT-PCR was performed on the ABI Prism 7500 Fast Sequence Detection System (Applied Biosystems, CA, USA). The 2^-ΔΔCt^ method was used to calculate and quantify the relative expression level of target genes. The following primers were used in this study: LOX-1 (F: 5’-TGATAGAAACCCTTGCTCG-3’, R: 5’- TTGCTTGCTGGATGAAGTC-3’); MCP-1 (F: 5’-GTTGGCTCAGCCAGATGCA-3’, R: 5’-AGCCTACTCATTGGGATCATCTTG-3’); CXCL-2 F: 5’-GGCAGAAAGCTTGTCTCAACCC-3’, R: 5’-CTCCTTCAGGAACAGCCACCAA-3’); eNOS (F: 5’-GTGGCTGTCTGCATGGACCT-3’, R: 5’-CCACGATGGTGACTTTGGCT-3’); Occludin (F: 5’-TACAGCAATGGAAAACCACACT-3’, R: 5’-CAAAGGAATGGGAAACGACTAA-3’); KLF2 (F: 5’-CCAAGAGTTCGCATCTGAAGGC-3’, R: 5’-CCGTGTGCTTTCGGTAGTGGC-3’); GAPDH (F: 5’-GACGGCCGCATCTTCTTGT-3’, R: 5’-CAGTGCCAGCCTCGTCCCGTAGA-3’).

### Western blot assay

HUVECs were lysed with the RIPA lysis buffer (Beyotime, Shanghai, China) containing protease and phosphatase inhibitor cocktail (Roche, Shanghai, China) followed by separating the proteins with the 12% SDS-PAGE. The separated proteins were then transferred to the polyvinylidene fluoride (PVDF) membranes (Thermo Fisher Scientific, MA, USA) using a semi-dry transferring method. The non-specific binding proteins were removed by incubation with the 5-10% BSA solution for 1-2 hours, followed by being incubated with the primary antibodies against LOX-1 (1:1000, Abcam, MA, USA #60178), eNOS (1:1000, Abcam, #ERP19296), Occludin (1:1000, Abcam, #ERP8208), KLF2 (1:1000, Abcam, #ab236507) or GAPDH (1:1000, Abcam, #ab8248) overnight at 4° C. After being incubated with the horseradish peroxidase-conjugated secondary antibody (1:3000, Abcam, MA, USA) for 1.5 hours at room temperature, the blots were developed with the ECL reagents (Amersham, UK) and exposed under Tanon 1600R (Tanon, Shanghai, China).

### ELISA assay

The concentrations of MCP-1 and CXCL1 in HUVECs were determined using the commercial ELISA assay kits (R&D System, MN, USA, #DCP00; #DY276-05). Briefly, the cell lysis sample was added to the wells of the 96-well plate and incubated with 1% BSA in PBS to remove the non-specific binding. The primary antibodies were also added for incubation overnight, followed by being washed using TBST. Subsequently, the samples were mixed with avidin conjugated to horseradish peroxidase (HRP). Following incubation with 3,3′,5,5′-tetramethylbenzidine (TMB) substrate solution, the absorbance of each well at 450 nm was detected using the spectrophotometer (Thermo Fisher Scientific, MA, USA).

### DAF-FM DA staining

DAF-FM DA dye (Thermo Fisher Scientific, MA, USA, #D23844) staining was used to assess the production of NO from treated HUVECs. In brief, the cells were plated on the 12-well plates and serum-starved with EBM for 16 hours, followed by incubation with 2.5 μM DAF-FM DA solution at 37° C for 30 minutes. Fluorescent signals were visualized using a fluorescent microscope.

### Endothelial monolayer permeability with FITC-dextran permeation

The *in vitro* vascular permeability assay kit from Millipore™ (Millipore, Paris, France) was used to determine the permeability of the HUVECs monolayer. Briefly, the cells were planted on the collagen-coated semipermeable inserts at a density of 3 × 10^5^ cells/insert for 48 hours, followed by replacing the medium with fresh cultural medium containing FITC at a ratio of 1:40. After incubation for 20 minutes in the dark, the absorbances were detected with a TECAN™ spectrophotometer at 485–535 nm.

### Statistical analysis

Mean ± standard deviation (SD) was used to represent the results. Analysis of variance (ANOVA) followed by Tukey’s post-hoc test was utilized for the contrast among different groups. All statistical analysis was analyzed using GraphPad software (version 6.0, GraphPad Software). *P*<0.05 was regarded as a statistically significant difference.
